# Sexual segregation results in pronounced sex-specific density gradients in the mountain ungulate, *Rupicapra rupicapra*

**DOI:** 10.1038/s42003-023-05313-z

**Published:** 2023-09-25

**Authors:** Hendrik Edelhoff, Cyril Milleret, Cornelia Ebert, Pierre Dupont, Thomas Kudernatsch, Alois Zollner, Richard Bischof, Wibke Peters

**Affiliations:** 1 Wildlife Biology and Management Research Unit, Bavarian State Institute of Forestry, Freising, Germany; 2 Faculty of Environmental Management and Natural Resource Management, Norwegian University of Life Sciences, Ås, Norway; 3 Seq-IT GmbH & Co.KG, Department Wildlife Genetics, Kaiserslautern, Germany; 4 Department of Conservation and Biodiversity, Bavarian State Institute of Forestry, Freising, Germany; 5 Wildlife Biology and Management Unit, Technical University of Munich, Freising, Germany

**Keywords:** Behavioural ecology, Ecological modelling

## Abstract

Sex-specific differences in habitat selection and space use are common in ungulates. Yet, it is largely unknown how this behavioral dimorphism, ultimately leading to sexual segregation, translates to population-level patterns and density gradients across landscapes. Alpine chamois (*Rupicapra rupicapra r*.) predominantly occupy habitat above tree line, yet especially males may also take advantage of forested habitats. To estimate male and female chamois density and determinants thereof, we applied Bayesian spatial capture-recapture (SCR) models in two contrasting study areas in the Alps, Germany, during autumn. We fitted SCR models to non-invasive individual encounter data derived from genotyped feces. Sex-specific densities were modeled as a function of terrain ruggedness, forest canopy cover, proportion of barren ground, and site severity. We detected pronounced differences in male and female density patterns, driven primarily by terrain ruggedness, rather than by sex-specific effects of canopy cover. The positive effect of ruggedness on density was weaker for males which translated into a higher proportion of males occupying less variable terrain, frequently located in forests, compared to females. By estimating sex-specific variation in both detection probabilities and density, we were able to quantify and map how individual behavioral differences scale up and shape spatial patterns in population density.

## Abstract (web summary)

Non-invasive fecal genotyping on Alpine chamois (*Rupicapra rupicapra*) in combination with spatial capture recapture models reveal sex-specific density patterns, which are driven by environmental features.

## Introduction

Far from being homogeneous, wildlife populations are composed of individuals that vary in their morphology, physiology, and behavior^1–3^. These intraspecific differences may scale up to population-level patterns in the configuration and dynamics of populations. One common source of such variation with population-level consequences is gender differences in life history and behavior^4,5^. Sexual behavioral dimorphism is particularly pronounced in ungulate species due to different strategies by both sexes to maximize their fitness (i.e., lifetime reproductive success), such as territoriality or forage-safety trade-offs, often leading to some degree of spatial segregation between sexes in the same population^6–8^. Despite a keen interest in the spatial distribution patterns of ungulates, an ecologically, culturally, and economically important taxonomic group, we lack quantitative information about how sex-specific determinants of individual space use within the landscape translate to population-level patterns in density. To this day, scaling up from a limited number of individual-based observations to population-level inferences remains an ubiquitous challenge in ecology^9,10^.

Estimating population parameters such as density is particularly difficult in mountain ungulates due to the rugged and remote terrain they occupy^11^. In open alpine areas, where sightability is sufficient, at best, relative abundance can be obtained by direct counts. However, this becomes even more challenging for ungulates that occur in forests, where visibility is limited and direct counts are not feasible^12–14^. Female and male chamois (*Rupicapra spp.)*, an ungulate inhabiting mountainous areas in Europe and southwest Asia, are known to exhibit different habitat selection patterns within the same population^15,16^. Although morphological sexual dimorphism in chamois is low compared to most ungulates species, male and female chamois employ contrasting strategies to maximize their fitness, which results in diverging behaviors^17^, differences in habitat use and sexual segregation for most of the year^18–20^. While females focus on increasing offspring survival and primarily occur in groups^21^, males commonly roam alone or in smaller groups. Sexual segregation is facilitated further by male mating tactics as some males actively defend territories, selecting lower elevations and potentially forested habitat throughout the warm season in contrast to non-territorial males which tend to follow female groups^19,22^. These behavioral differences often result in a wider altitudinal range covered by male chamois compared to females^23^. To date, inferences on sex-specific differences in space use in chamois are primarily based on telemetry studies which typically rely on a small fraction of tagged individuals in a population^15,24^ or on distributions and relative abundances derived from direct observations, which are commonly limited to habitats above tree line with good visibility^18^. Since habitat use in chamois has been shown to be very plastic, comprising also montane and subalpine forests^25^ where limited visibility is a detriment to detectability of animals during direct observation surveys^26^, deriving population-level inferences has been challenging. Consequently, it is unknown how potential differences in habitat selection and spatial segregation between sexes translate to distributional differences and density gradients at the population level.

In this study, we aimed to identify how population-level patterns in Alpine chamois (*R. r. rupicapra*) density emerge from sex-specific environmental preferences. We combined non-invasive genetic sampling (NGS) with spatial capture-recapture (SCR) models to estimate overall and sex-specific densities of Alpine chamois in two study areas in the Bavarian Alps, Germany. This approach offers population-level insights by accounting for imperfect detection^27,28^ and estimating the link between density and spatial determinants thereof^29^. The SCR approach takes advantage of the information contained in the spatial configuration of individual detections to link abundance estimates with spatial distribution^27,28^. By using spatial covariates on density, SCR further allows estimation of habitat preferences at the population level and map population-level patterns in density^29^.

Herein, we identified the sex-specific influence of habitat characteristics and describe how patterns in male and female habitat selection scale up to shape the density surface of two chamois populations. After selecting potential habitat variables driving the density gradients of male and female chamois, we tested for the role of four key determinants of chamois density distribution, namely terrain ruggedness, canopy cover, site severity as a proxy for thermal conditions, and the amount of barren ground. We predicted that the different space use strategies of males and females would manifest in contrasting density surfaces. Specifically, we expected the stronger focus on foraging opportunities and safety by females to lead to a pronounced association with rugged areas which are often located above the tree line in our study areas, therefore resulting in comparatively higher densities of females at high elevation. In contrast, we expected an overall more homogenous distribution of males due to a stronger plasticity in habitat selection. We also expected males to prefer canopy cover as territorial males tend to occupy areas around or below the tree line^19,23^. Barren ground is low in forage, potentially resulting in a negative effect on chamois densities. Regardless, we expected the effect of barren ground to be weaker for females than for males, because, during the vegetative period, female groups tend to take advantage of areas above tree line where barren ground is also more prominent, but may be interspersed with escape terrain and forage-rich habitat such as alpine meadows^17^. Finally, while chamois have been shown to select for south-facing slopes irrespective of sex or season^17^, especially towards the end of the vegetative period, we expected a slight preference for mesic (north-facing) over xeric (south-conditions) sites^30^, due to trade-offs between forage availability, thermoregulation and reproductive strategies^23^.

## Results

### Field sampling and genetic analysis

We performed systematic feces collections within each study area to obtain individual detections from NGS data and derive conceptual traps for the SCR models. In total, longer search paths (817.58 km) were performed in Chiemgau (CG), which resulted in 5663 conceptual traps and 465 fecal samples collected. In Karwendel (KW), 458.96 km of search paths were recorded, which produced 3546 conceptual traps covering the study area and 1384 fecal samples. For KW, 1193 of the 1384 samples yielded a consensus genotype suitable for further analyses. For CG, 259 of the 465 samples resulted in useable consensus genotypes. Overall, more individuals (*n* = 616, *n*_female_ = 301, *n*_male_ = 292, *n*_NA_ = 23) were identified in KW compared to CG (*n* = 154, *n*_female_ = 102, *n*_male_ = 52). Reliable individual discrimination was indicated by a probability of identity (PID) of 4.1 × 10^−8^ for the KW and 5.3 × 10^−7^ for the CG dataset, and a PID for siblings (PIDsib) of 0.0010 for the KW and 0.0022 for the CG data set. The mean number of detections per individual derived from the conceptual traps was 1.67 (sd = 1.04) in KW and 1.50 (sd = 0.89) in CG. The rate of detections also varied by sex and was 1.75 (sd = 1.15) for males and 1.64 (sd = 0.95) for females in KW and 1.33 (sd = 0.81) for males and 1.59 (sd = 0.92) for females in CG (more details on detections provided in Supplementary Table 1).

### Detection probability

Based on locations of genotyped fecal samples we derived individual encounter histories and estimated detection probabilities conditional on locations of individual activity centers (AC). First, in both study areas, detection probability increased with search effort (95% Bayesian Credible Intervals are provided in brackets; CG: ${\beta }_{{{{{{{\rm{search}}}}}}}}$ = 0.631 [0.528–0.736]; KW: ${\beta }_{{{{{{{\rm{search}}}}}}}}$ = 0.579 [0.523–0.636]; Supplementary Table 2). Next, baseline detection probabilities (${p}_{0}$) of both sexes were higher in KW (${p}_{{0}^{{{{{{{\rm{female}}}}}}}}}$ = 0.013 [0.010–0.015]; ${p}_{{0}^{{{{{{{\rm{male}}}}}}}}}$ = 0.025 [0.021–0.030]) compared to CG (${p}_{{0}^{{{{{{{\rm{female}}}}}}}}}$ = 0.005 [0.003–0.007]; ${p}_{{0}^{{{{{{{\rm{male}}}}}}}}}$ = 0.003 [0.001–0.005]). There was no significant difference in the scaling parameter ($\sigma$), which can be seen as a proxy for home range size, between males and females in CG (*σ*_female_ = 278 m [247–314 m]; *σ*_male_ = 280 m [220–367 m]), whilst it was 35% smaller for males compared to females in KW (*σ*_male_ = 150 m, [139–160 m]; *σ*_female_ = 227 m, [211–244 m]).

### Abundance and density estimation

Abundance estimates derived from the SCR model yielded 186 [153–227] females and 134 [93–198] males in CG which translates to a skewed overall sex ratio towards females (${\varPsi }_{{{{{{{\rm{sex}}}}}}}}$ = 0.39 [0.27 - 0.53]; i.e., male:female ratio approximates 0.72; Table 1). In KW, abundance estimates were 506 [446–574] females and 510 [456–572] males, which was slightly skewed towards males (${\varPsi }_{{{{{{{\rm{sex}}}}}}}}$ = 0.56 [0.50–0.61]); male:female ratio approximates 1.01). In general, the average density was lower in the more forested study area. Specifically, average density was almost five times lower in CG ($\hat{D}$ = 4.09/km^2^ [3.38–5.03]) compared to KW ($\hat{D}$ = 19.37/km^2^ [17.84–21.08]).

**Table 1:** Estimated Chamois densities and population sizes derived from the SCR models.

Study Area	Group	$\hat{{{{{{\rm{N}}}}}}}$	Density	$\hat{{{{{{\rm{N}}}}}}}$ (forest)	Density (forest)	$\hat{{{{{{\rm{N}}}}}}}$ (open)	Density (open)
CG	Female	186 (153–227)	1.71 (1.19–2.53)	75(55–99)	1.35 (0.99–1.79)	111 (86–141)	4.89 (3.79–6.21)
	Male	134 (93–198)	2.38 (1.96–2.91)	68 (42–107)	1.23 (0.76–1.94)	66 (41–105)	2.91 (1.81–4.63)
	Overall	320 (264–393)	4.09 (3.38–5.03)	143 (109–187)	2.58 (1.97–3.38)	177 (139–225)	7.80 (6.13–9.92)
KW	Female	506 (456–574)	9.65 (8.51–10.95)	113 (95–134)	3.39 (2.84–4.00)	392 (339–454)	21.07 (18.20–24.38)
	Male	510 (456–572)	9.73 (8.70–10.91)	166 (143–191)	4.94 (4.27–5.70)	344 (300–395)	18.49 (16.11–21.21)
	Overall	1016 (935–1105)	19.37 (17.84–21.08)	279 (250–310)	8.31 (7.46–9.25)	737 (666–814)	39.56 (35.75–43.70)

Posterior means and 95% Bayesian credible intervals. Study Area CG = Chiemgau, KW = Karwendel. $\hat{N}$ estimated number of individuals. Density individuals/km^2^. Estimates derived for each entire study area and within forested and open regions of each study area based on a post-hoc analysis using the results of the SCR models.

### Determinants of densities

**Fig. 1:**
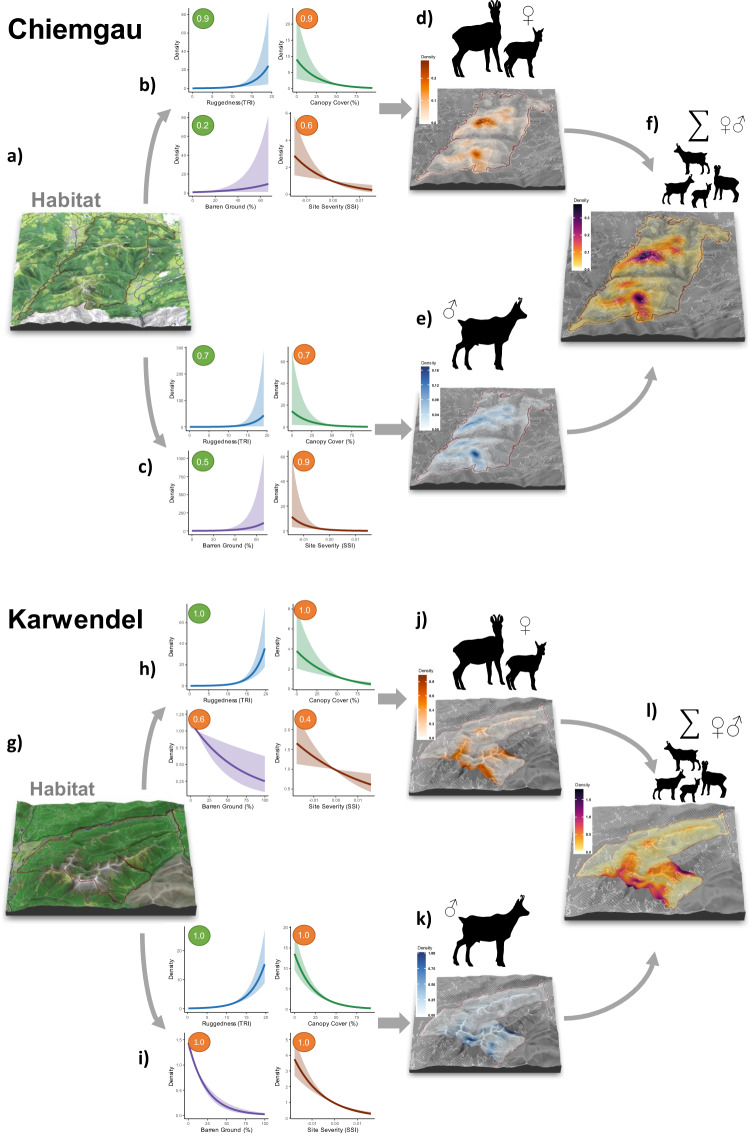
Environmental determinants of population and sex-specific density surfaces of Alpine chamois in two study areas in Germany. Maps representing the habitat configuration (**a**, **g**) and spatial distributions of sex-specific (**d**, **e**, **j**, **k**) as well as overall (**f**, **l**) chamois densities resulting from the Bayesian SCR models for the two study areas Karwendel and Chiemgau. Values shown in the density maps represent average numbers of individuals/ha retrieved from posterior samples. Effects of changes in single covariates (response curves for ruggedness, site severity, canopy cover and barren ground depicted in **b**, **c**, **h**, **i**) on the intensity of the point process were derived keeping the mean value of the three other covariates. Circles indicate variable importance (number = posterior inclusion probability) and direction of the coefficient value (green = positive, orange = negative). Maps and response curves were produced using the R software. Maps are based on data derived from a digital elevation model^79^ and a digital land cover model (ATKIS data^81^). Chamois silhouette adapted from image by Ferran Sayol (CCO 1.0 Public Domain; www.phylopic.com).

Our results showed spatially heterogenous densities for both sexes (Table 1, Fig. 1). We tested for the effects of four explanatory covariates (ruggedness = TRI; canopy cover = CANOPY, barren ground = BARREN, site severity = SSI) on the ecological process describing the placement of ACs. Bayesian multimodel inference allowed us to derive posterior inclusion probabilities (PIP, Table 2) for each variable and overall posterior model probabilities (PMP, Supplementary Table 3). Distribution of ACs of both sexes in KW were best described by a full model (M_15_) including all four covariates (PMP_females_ = 39%, PMP_males_ = 100%; Supplementary Table 3). In contrast, densities of female and male chamois in CG were primarily driven by the model comprising TRI, SSI and CANOPY (M_12_: PMP_females_ = 44%, PMP_males_ = 50%; Supplementary Table 3).

**Table 2:** Posterior variable inclusion probabilities and coefficients for the four covariates considered in the SCR model to estimate chamois densities.

Study Area	Sex	Covariate	PIP	Coefficient	CI low	CI high
CG	Female	TRI	0.99	1.18	0.56	1.64
		SSI	0.61	−0.40	−0.68	−0.13
		CANOPY	0.95	−0.98	−1.41	−0.50
		BARREN	0.22	0.19	0.02	0.37
	Male	TRI	0.72	1.37	0.37	2.10
		SSI	0.99	−0.93	−1.58	−0.44
		CANOPY	0.69	−1.19	−1.90	−0.29
		BARREN	0.49	0.39	0.06	0.59
KW	Female	TRI	1.00	1.55	1.25	1.86
		SSI	0.39	−0.22	−0.39	−0.05
		CANOPY	1.00	−0.62	−0.98	−0.34
		BARREN	0.67	−0.29	−0.50	−0.09
	Male	TRI	1.00	1.18	0.95	1.43
		SSI	1.00	−0.58	−0.73	−0.44
		CANOPY	1.00	−1.21	−1.38	−1.05
		BARREN	1.00	−0.80	−0.98	−0.63

*Study Area* *CG* Chiemgau, *KW* Karwendel. *PIP* posterior variable inclusion probabilities of the covariates considered in the spatial point process of the SCR model. *Coefficient* model averaged coefficient estimates (posterior values when variable was included in the model). *CI* 95% Bayesian credible intervals.

**Fig. 2:**
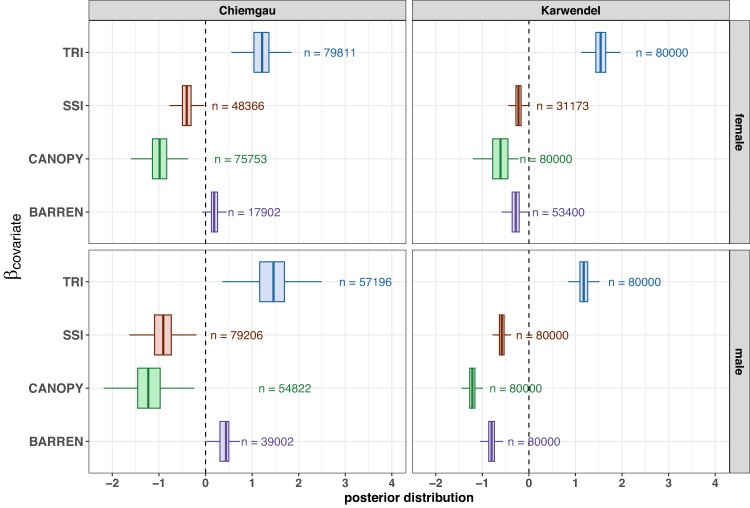
Posterior probability distribution of coefficients describing the relationship between chamois density and habitat covariates estimated by the Bayesian SCR model. Coefficients are derived from posterior samples for iterations when the rjMCMC variable indicated that the habitat variable was included in the model (n = number of iterations with indicator variable γ = 1 and used in the boxplots). Boxplots depict the 1.5 interquartile range (whiskers), lower and upper quartiles (hinges) as well as median values of each distribution.

The coefficient estimates and PIPs > 70% (Table 2) indicated that the distribution of ACs was positively influenced by TRI for both sexes and study areas, confirming that terrain ruggedness is an important predictor of chamois density, irrespective of sex (Fig. 2). Canopy cover was included in > 90% of the iterations for females in CG and both sexes in KW, confirming an overall high influence of this variable on chamois density (Table 2). Coefficient estimates for canopy cover consistently showed a negative effect on AC placement (Fig. 2). The influence of barren ground on density was inconsistent for the two study areas. In CG, this covariate was less frequently included in the models (PIP_females_ = 22%, PIP_males_ = 49%) and its effect was small for males (*β*_3,male_ = 0.39 [0.06–0.59]) and even lower for females (*β*_3,female_ = 0.19 [0.02–0.37]) when they were included, suggesting little evidence for an influence on chamois densities in this study area. In contrast, barren ground was included in >60% of the models in KW (Table 2) and parameter estimates (*β*_3,female_ = −0.28 [−0.49 to −0.09]; *β*_3,male_ = −0.80 [−0.98 to −0.63]) suggested a negative effect, especially for males, in this study area.

Xeric site conditions (i.e., SSI) had a negative effect on the density of both sexes. However, the influence of SSI on AC placement was larger for males (PIP > 0.99) than for females (PIP < 0.61) in both study areas (Table 2). Also, coefficient estimates for females were closer to zero whereas for males in both study areas coefficient estimates were significantly negative (CG: ${\beta }_{4,{{{{{{\rm{male}}}}}}}}=$ −0.93 [−1.58 to −0.44]; KW: ${\beta }_{4,{{{{{{\rm{male}}}}}}}}=$ −0.58 [−0.73 to −0.44]).

A post-hoc analysis to derive the number of individuals located within and outside of forested areas supported our prediction that sex-specific differences in density would be particularly pronounced in forests. We found a higher proportion of males in forested areas (33% in KW, 51% in CG) than females (22% in KW, 40% in CG). In general, less than 30% of chamois ACs were located in forests in KW, compared to 45% in CG (Table 1), suggesting regional effects related to general habitat availability.

## Discussion

Our study revealed markedly different autumn density surfaces for female and male chamois within the same population. These population-level patterns were linked to sex-specific responses to the configuration of the landscape. Pronounced and contrasting sex-specific spatial patterns in density emerged even though sex differences were quantitative rather than qualitative. Female densities appeared to be higher in areas with more escape terrain (primarily characterized by ruggedness), whereas male densities were predominantly driven by a combination of canopy cover and escape terrain. Site severity, on the other hand, emerged as relevant only for male density distribution in KW.

While we were able to provide not only chamois densities, but also gradients thereof, the scope of our estimates and the associated effects of covariates are restricted to the timing of our field surveys, i.e., autumn. Sex-specific differences in density were especially pronounced in Karwendel, the study area with a more complex alpine landscape, i.e., more rugged terrain above tree line and higher overall chamois densities. Female spatial behavior is less constrained by reproductive needs than male spatial behavior, and more by the need to raise and protect offspring and gain access to high-quality food resources^6,17,23^. Consistent with this, we found that females exhibited noticeably higher densities in non-forested regions (e.g., above tree line) when such habitat was available. During the time of our field surveys females are still focused on maternal care and try to capitalize upon the extended growth season along topographically diverse mountain slopes before moving down when snow makes living at higher elevations difficult^15,20^. In contrast, different reproductive strategies co-exist in male chamois, that may affect the extent to which forested habitats are used throughout the year^23^. Territorial males tend to inhabit areas at lower elevations that will remain snow-free during the rutting season and are therefore attractive to females^19,23,24^. Non-territorial males track the upward shift in vegetation phenology during summer to take advantage of better foraging opportunities at higher elevations and display habitat selection patterns that are comparable to those of females^23^.

Several authors have shown that adjustments in behavior, such as habitat selection, are important mechanisms by which mountain-dwelling species buffer the effect of climate change to some extend^31,32^. In terms of site severity, males showed higher preference for mesic sites in both study areas. This effect was in the same direction, although weaker, for females. Stronger selection for sites that are moister and less sun-exposed by chamois may be attributed to reduced heat-stress and a prolonged growing period in autumn in these areas compared to more xeric sites^30^. Further, chamois may also shift between southern and northern exposures at a smaller spatio-temporal scales that we were not able to address with the resolution of our data, e.g. within their home range during the course of a day^33^.

Sexual differences were evident not only in the effect of environmental covariates on density distributions, but also in the spatial extent of home ranges. Population-level inferences about the latter are possible in SCR due to the link between the detection function’s scale parameter (σ) and the related movement around an individual’s center of activity^34^. However, direct comparisons with results from e.g. telemetry studies are only possible in terms of relative differences rather than absolute values due to the much lower frequency of relocations (individual recaptures) and simplified movement assumptions of the SCR model^27,35^. Nevertheless, the results (both from σ estimates and observed distances between samples, see Supplementary Tables 1 and 2) can be explained ecologically. Our analysis revealed larger home range sizes (derived from the σ estimates) for females than for males, at least in KW. As part of another study, we also observed similar patterns in home ranges derived from telemetry data in this area (see Supplementary Fig. 1). Boschi and Nievergelt^36^ also found smaller home ranges in male compared to female chamois. Female chamois commonly live in groups^21^ and consequently require larger amounts of high-quality forage resources per group compared to solitary-living males^37^. This can explain why female home ranges tend to be bigger than those of males, especially during the vegetation period^15,36^. In contrast, both sexes showed longer movements in CG (Supplementary Table 1), the study area at the edge of the species distribution. This could be caused by the limited amount of alpine habitat in this area resulting in similar constraints on habitat use regarding resources and safety for both sexes. The inverse relationship between habitat quality and home range size, and hence movement, has already been shown for several other ungulate species^38^.

Differences in environmental context and landscape configuration in the two study areas modulated our findings. As expected, chamois showed a strong preference for rugged terrain, but density patterns varied greatly as a function of habitat availability. For example, the role of barren ground differed between study areas and could be an indication of a functional response^39^ to the low availability (approximately 3%) of this habitat type in CG (positive effect) in contrast to KW (negative effect) where this habitat type covers about 16% of the study area, mainly above tree line. While representing potential escape terrain, these areas provide only limited forage. In areas with strong human impact like tourism and hunting, as is the case in CG, the choice of such habitats could also reflect greater emphasis on safety.

We observed only slightly divergent associations of male chamois with canopy cover between the two study areas. In KW most male ACs were positioned around the tree line due to the combination of selection for rugged terrain and avoidance of regions with higher levels of canopy cover or barren ground. Such border regions may provide both advantages of open and closed habitats and allow male chamois to trade-off between limiting factors such as mating opportunities, forage, and possibly hunting risk at different spatio-temporal scales (e.g., Dupke et al.^40^). This preference for the tree line could result from the presence of both space-use strategies (territorial vs. non-territorial) in the population at the onset of autumn shortly before the upcoming rut (end of November until mid-December). If that was the case, however, we would expect stronger association with canopy cover in CG as sampling in this study area was closer to the onset of the rut. Interestingly, we detected no such effect of canopy cover on male AC placement in CG. An additional analysis using external telemetry data from radio-collared chamois (*n* = 16) in Karwendel revealed that male chamois habitat preferences remain stable throughout autumn until the beginning of the rut (Supplementary Note 1, Supplementary Figs. 1, 2).

While SCR combined with non-invasive genetic sampling is now relatively frequently used for the study of large carnivore populations, it is still rarely applied to ungulates^27^. Our approach allowed us to achieve a scale-transcending perspective from individuals to the population level, since SCR also approximates the second-order (landscape scale) process of habitat selection^41^ (also see Supplementary Note 1). We thereby overcame shortcomings typically plaguing studies on sexual segregation that generally draw their inferences on a subset of individuals using e.g., radiotelemetry or direct observations and the environmental conditions experienced by those individuals. Even if the study areas could not be searched in their entirety, it has been shown that SCR analyses are robust to spatial gaps in sampling, as long as individuals with an AC inside these gaps can potentially be detected in the surrounding detector grid cells^42^. In our case, individuals utilizing the non-accessible and therefore unsearched areas were likely detectable in the searched grid cells. Overall, we applied a high-resolution sampling design resulting in a dense grid of detectors, especially in comparison to the space use and ranging behavior of our study species, to maximize the probability of detecting individuals at multiple locations^43,44^.

We considered a broad range of habitats from far below to above the tree line and found that a considerable proportion of chamois in both study areas were located in forested areas during autumn. Without correcting for imperfect detectability or sightability, traditional approaches like block counts^26^ would most likely underestimate true abundance and the magnitude of sexual segregation in habitat use. It is therefore not surprising that our density estimates correspond to either average (CG) or high (KW) densities when compared to the literature. Similarly, the reported sex ratios might be affected by the adopted census methodology. Individuals inhabiting forests are difficult to count and block surveys are therefore more prone to underestimate their proportion of the overall population. Therefore, if males are more likely to be located in forested areas their proportion could be underestimated^26,45^.

We detected a female-biased sex ratio in CG and a slightly male-biased ratio in KW. Reasons for the observed uneven sex ratios can be manyfold and related to differential hunting pressures on distinct sex/age classes, natural mortality, or poaching. We also do not know if the sex ratio reported here is representative across all age classes, since all age classes are combined in our analyses. The sex ratio of the adult population could hence differ slightly from the overall ratios that emerged from our SCR analyses. In general, besides gender, age is an important source of heterogeneity in behavior and habitat use in ungulates^46^. Although age cannot be determined genetically and thus currently remains unaccounted for in NGS-based SCR analyses, a recent simulation study indicated that bias caused by unaccounted variability in space use related to age only produces minor deviations in density estimates^47^. Overall, in chamois this is more likely an issue for males which exhibit stronger behavioral plasticity and potentially habitat preferences depending on age-class (juvenile vs. adult) compared to females which mostly move in groups^21^.

Numerous simulation studies indicate strong robustness of density estimates derived from SCR models towards violations of model assumptions such as deviations of true space use of individuals from homogenous or circular home ranges^34,48–51^. While we were able to account for two major sources of variability, namely sex-specific differences in detectability^52,53^ and density, unaccounted heterogeneity within the encounter data and small sample sizes (limited number of individuals with multiple detections) can potentially introduce a negative bias to density estimates and yield artificially high precision^54–56^. With larger sample sizes and more recaptures, latent individual heterogeneity could be quantified by fitting an SCR model including a mixture component on the baseline detection probability^56^. These type of mixture models are also commonly used in non-spatial capture recapture models^34,57^. Not accounting for individual heterogeneity in detectability and space use may lead to an underestimation of population size^42,58^. However, this bias becomes negligible when individual variability within the population is relatively small^47^.

The observed patterns in density in our study may also have important management implications such as the identification of key areas relevant for population or habitat management, including refuge habitat for wildlife or sites where high densities interfere with management goals^59^. For example, recent studies suggest a shift of alpine chamois towards habitats that provide cover and thereby enhance thermoregulation in response to climatic changes and rising temperatures^31,60^. Due to such shifts, increases in density especially at the edge of the distribution in areas with lower elevations below tree line (e.g. CG) may be possible in the future, potentially leading to new management challenges associated with chamois in the Alps with forestry due to overbrowsing^59^. Mountain ungulates are particularly constrained in their distribution, and their habitat has been subjected to rapid change in human land use practices and climate^61,62^. This raises concerns about current and future distributions and densities^63^. Populations located at the edge of their distribution range tend to be affected disproportionately as they experience marginal conditions in terms of environmental suitability and a shift in the tug-of-war between resources and constraints^64^. Therefore, information about the habitat-density relationships derived at the population level can help inject much-needed factual information into debates on management challenges associated with chamois in the Alps^65^. Here, we showed that non-invasive genetic monitoring in combination with SCR provides a suitable monitoring tool to periodically assess and track changes in several ecologically meaningful parameters while accounting for differences between sexes.

## Methods

### Study areas

**Fig. 3:**
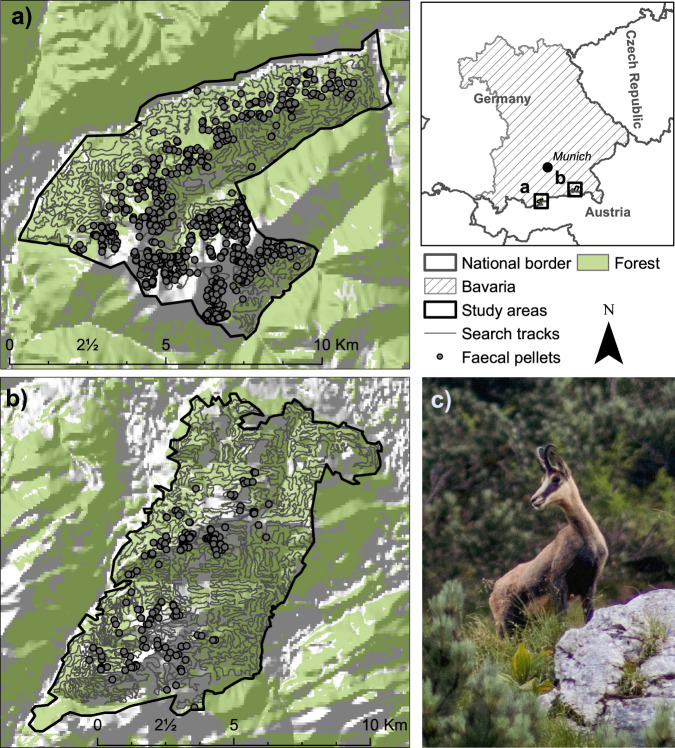
Overview of Alpine chamois study areas in Bavaria, Germany. **a** Map of the Karwendel study area including fecal pellet locations, search tracks and forest landcover. **b** Map of the Chiemgau study area including fecal pellet locations, search tracks and forest landcover. **c** image of an Alpine chamois individual from the Karwendel study area (Image Credit: Bavarian State Institute of Forestry, LWF). Maps are based on data derived from a digital elevation model^79^ and a digital land cover model (ATKIS data^81^). All maps were produced using the ArcMap™ software licensed through Esri.

This study was conducted in two mountainous areas in the Bavarian Alps, Germany, both immediately bordering Austria (Fig. 3). The “Karwendel” study area covers approximately 5250 ha and is located in the correspondent Karwendel mountain range. Elevation ranges 800 m to approximately 2350 m above sea level. The second study area, “Chiemgau”, covers about 7250 ha and elevation ranges from 600 m to roughly 1800 m above sea level. Both study areas are composed of typical central European alpine forest communities including European beech (*Fagus sylvatica*), Norway Spruce (*Picea abies*), silver fir (*Abies alba*), larch (*Larix spp*.) and sycamore (*Acer pseudoplatanus*), with mountain pine (*Pinus mugo*) forming krummholz complexes at higher elevations in open and steep terrain. Forests cover about 60% and 70% of the KW and CG study area, respectively. Non-forested regions are dominated by either alpine meadows and pastures or barren ground covered by rocks and debris/boulders. Open landscapes are mainly characterized by pastures grazed by cattle during the summer months, especially in CG. While both areas are partially (CG) or completely (KW) nature reserves, they are under substantial human influence due to tourism, hunting, and other land use practices. Human impact is higher in CG. For example, the combined density of forestry roads and hiking trails is 3.1 km/km^2^ in KW, while it is 4.9 km/km^2^ in CG. Chamois have few natural predators in both study areas, except Golden eagles (*Aquila chrysaetos*) and foxes (*Vulpes vulpes*) which occasionally predate kids. Hunting is likely the main cause of mortality in both study areas, but rates of natural mortality are unknown. Hunting is permitted according to federal and state hunting regulations throughout most of the study areas from August 1st to December 15th. Exceptions are permitted in restricted regions to prevent browsing of mountain forest regeneration. Averages of harvested animals/area unit approximate 2.4 chamois harvested/km^2^ in KW and 1.6 chamois harvested/km^2^ in CG with the harvest peak in August (average of 2011–2018). The ungulate guild is comprised of red deer (*Cervus elaphus*) at comparable densities in both study areas, while roe deer (*Capreolus capreolus*) are more common in CG.

### Non-invasive genetic sampling

Individual detections were obtained through non-invasive fecal sampling based on a systematic survey design. Field surveys were performed across the entire elevation gradient, and both forested as well as open landscapes within our study areas. Sampling took place within a three-week period for each study area in autumn 2018 (September 24th until October 7^th^ in KW and October 4th to 29th in CG). We superimposed a 200 m search grid onto each study area and pooled up to 16 grid cells into daily search units. We performed unstructured searches for fresh fecal piles within each of these grid cells by teams of two observers. Only fresh feces with intact pellets and moist, shiny surfaces were collected and their spatial location recorded with GPS devices (mainly Garmin eTREX 10 and Garmin 65 series). All search teams were instructed to cover the assigned grid cells with comparable intensity but dangerous and inaccessible terrain (e. g. too steep) was excluded. All search tracks were recorded with GPS devices to later account for spatially heterogenous search effort. Fecal samples were stored in 50 ml falcon tubes frozen at the end of each sampling day at -20° C. No ethical approval was required as the study only involved non-invasive genetic samples from feces and no direct capture or handling of wild animals. No permits were required to carry out the field work and obtain feces samples but field work was coordinated with management authorities.

### Genetic analyses

For isolation of DNA, we used a commercial kit (NucleoSpin Soil Kit, Macherey-Nagel, Baesweiler, Germany). To achieve a high proportion of target DNA, two pellets of each sample were washed with 1.5 ml lysis-buffer at room temperature in a 50 ml Falcon tube, thereby avoiding the destruction of the pellets and including mainly the mucosal layer on the pellet surface that contains most of the intestinal cells. For individual identification of chamois, we selected eight microsatellite markers already tested for chamois based on their variability and suitability for multiplexing as well as their performance with DNA extracted from feces (Supplementary Table 4). We aimed to achieve a probability of identity for siblings (PIDsib $\ll$ 0.01) to ensure reliable individual discrimination^66^. The eight microsatellites were combined in two separate multiplex PCRs. For sex determination, we used a x- and y-chromosome-specific region of the Amelogenin gene according to Gurgul et al.^67^ which we integrated in Multiplex A (Supplementary Table 4). The thermocycling profile was as follows: 95 °C for 15 min, 45 cycles of 94 °C for 30 sec, 57 °C for 90 sec, and 72 °C for 60 sec, then 60 °C for 30 min. Amplification reactions were first performed as two replicates in a total volume of 12 μl each using the Qiagen Multiplex PCR kit (Qiagen, Hilden, Germany). The primers were used at concentrations of 0.1 μM to 0.4 μM. We separated fluorescently labeled DNA fragments on an ABI3730 DNA analyzer and determined allele sizes using the ABI GS500LIZ size ladder (Applied Biosystems, Darmstadt, Germany). We included two negative controls in every PCR to detect potential contamination. We repeated the genotyping another two times when results were ambiguous and deduced consensus genotypes from the results of the two to four replicates. Samples were typed as heterozygous at one locus if both alleles appeared at least twice, and as homozygous when all replicates showed the same result. All samples which failed to amplify or to produce unambiguous results for more than two loci were discarded. For samples with one or two missing loci, we re-checked raw data for plausibility in case of matching genotypes and excluded any sample matching with more than one genotype^68^. We also excluded samples showing signs of cross-contamination (i.e., genotypes with more than two alleles) from further analyses. For all analyzed samples, DNA extraction and PCR setup were carried out in separate rooms on different floors of the laboratory to avoid transfer of amplified DNA into the pre-amplification steps^69^. Determination of matching genotypes was carried out with GENECAP^68^. To confirm the power of the used loci, we calculated the probability of identity (PID) and, being more conservative, PID for siblings^66^ using GIMLET^70^.

### SCR modeling

We fitted single-season Bayesian SCR models with sex-specific parameters^34^ to estimate chamois density for each study area. Taking advantage of spatial encounter data (*sensu stricto* captures and recaptures), the SCR model is comprised of a) the ecological process which models the distribution of individual activity centers (ACs), and b) the detection process conditional on the distance to the AC of an individual. We assumed that both processes would vary among sexes and study areas and therefore estimated separate ecological and observation process parameters for females, males, and each study area.

The ecological process part of the SCR model allows for deriving local densities and testing for the effects of explanatory covariates on density^29^. As true ACs are unknown, they are treated as latent variables in SCR models. Spatial distribution of ACs can be modeled as the realization of a point process which is comparable to a second-order resource selection function^34^. Density estimates are then derived from the number of ACs located within a given area of interest. To allow for individual ACs to fall outside the sampled areas, we buffered each study area by 1000 m. We then placed a 250 m grid over the resulting area to define the habitat available for potential AC placement.

Without considering any covariates, the model represents a homogeneous point process in which ACs are distributed uniformly over the study area. Here, we used an inhomogeneous Bernoulli point process^71^ to explicitly account for the influence of habitat covariates on the distribution of ACs and therefore density. We chose a set of eight covariates which represent important determinants for sex-specific habitat selection of chamois at this particular scale (2nd order habitat selection^29,41^): information on geomorphology, habitat openness, composition of non-forested regions, and vegetation quality.

Regarding spatial variation in geomorphology, we considered elevation, terrain ruggedness, and site severity as potential covariates in the point process model. Being a proxy for several abiotic and biotic variables, including forest structure, temperature, and road density, elevation^72^ was identified as an important determinant of AC placement in chamois and male as well as female chamois have been shown to select for different altitudes throughout the year^15,23^. Further, heterogeneity in terrain composition may also be an important variable regarding the distribution of individuals since chamois are not strictly tied to high-elevation areas but also occur in steep regions at lower elevations^73^. In this regard, the terrain ruggedness index^74^ has been used as a proxy for topographic heterogeneity as well as potential escape terrain for mountain ungulates in previous studies^75^. To test how ACs are placed along the gradient of mesic (such as flat north facing slopes) to xeric (like steep south-facing slopes) sites, we consulted the site severity index^76^ which combines information on aspect and slope essentially serving as a proxy for both topographic position and thermal site conditions.

In addition to these orographic factors, the gradient between open to closed habitats, e.g. forests, may further impact sex-specific differences in the placement of ACs in chamois^15^. Therefore, we considered the degree of canopy closure as well as the distance to the nearest forest as potential covariates of the point process component. Non-forested regions (open habitats) in our study areas comprise differing amounts of barren grounds and alpine meadows which both have been shown to explain variation in home range placement of male and female chamois^17^. Hence, we derived the amount of as well as the distance to barren ground to characterize the composition of open habitats. Lastly, we considered a primary productivity index (the Normalized Difference Vegetation Index, NDVI) as a proxy for vegetation composition and quality especially outside of the closed habitats^23,77^.

All covariate values were initially retrieved at a resolution of 25 m and subsequently averaged over larger grid cells. Averaging the covariates allowed us to represent the overall conditions an individual would experience if its home range center was located in a given habitat cell (2nd order habitat selection^41^). Because home range size was larger in CG than in KW (as determined by σ estimates from preliminary analyses), we averaged the covariates within a 200 m radius in KW and a 300 m radius in CG. All covariates were then resampled to a 250 m habitat grid and standardized to a mean of zero and a standard deviation of one. We then tested for collinearity among the covariates (see also Supplementary Table 5) and removed one of two covariates when their pairwise correlation was high (|*r*| >  0.7)^78^.

The final set of covariates (see also Supplementary Fig. 3) included in the ecological process model consisted of the topographic ruggedness index (TRI), the site severity index (SSI), the percentage of canopy cover (CANOPY) as well as the percentage area covered by barren ground (BARREN). Both TRI and SSI were calculated based on a digital elevation model^79^. Topographic ruggedness was derived for each grid cell based on the differences in elevation with the immediately adjacent cells^75^. The site severity index (SSI) combines information on slope and aspect^76^ and served as a proxy for thermal site conditions ranging between values below zero (mesic sites) and above zero (xeric sites). To reflect the degree of habitat openness, we retrieved tree canopy cover (CANOPY) from remote sensing data source (*Copernicus*^80^). To characterize the amount of barren ground (BARREN), for example representing non- or sparsely vegetated rock walls, we used a digital land cover model (*ATKIS* data^81^). We quantified the relative importance of the final four covariates by applying a Gibbs sampling procedure^82^. This allowed for simultaneous computation of posterior model probability (PMP), the proportion of times a given combination of covariates was included, and posterior inclusion probabilities^83^ (PIP), the proportion of times a single variable was included in the model. Bayesian multimodel inference was performed accounting for all fifteen possible variable combinations. If none of the covariates were included, the model reduces to the null model and AC placement follows a uniform point process^29^.

Next, we modeled the individual detection probability conditional on its AC location to account for variation based on the distance to latent ACs. We used the locations of successfully genotyped fecal samples to derive individual encounter histories. We checked the distribution of observed distances between individual spatial recaptures for long distance outliers to avoid violations of the assumption of closed populations due to potential dispersal or migratory movements^84^. Recaptures with distances above the 99% percentile (longer than 1500 m) were removed. To fit the model to data from unstructured search tracks, we derived conceptual traps by placing a detector grid of 100 m cell size over the study areas^85^. Multiple detections of the same individual were partially aggregated into 50 m searched sub-grids allowing for up to four independent encounters within one of the main detectors^86^. Encounter frequency of an individual at each detector was therefore assumed to follow a binomial distribution with a maximum sample size of four. We modeled the decrease in detection probability with increasing distance between the AC of an individual and a detector based on commonly applied half-normal detection function where the level of decrease in the detection probability is described by a scaling parameter σ^34^. When applying the half-normal detection function, σ is proportional to the radius of a circular individual activity area^87^. To account for non-uniform search effort among detectors, we used a generalized linear model formulation to estimate the detector- and sex-specific baseline detection probability depending on the area searched within each detector grid cell. The area searched effectively within each detector was approximated by placing a 3 m buffer around each search track to approximate the viewshed of two observers. Grid cells that were not searched were not considered.

We estimated the number of undetected individuals by data augmentation^34,52^. For each study area, we set the maximum population size (*M*) to six times the number of identified individuals throughout the corresponding field survey. Whether an individual *i* from *M* is also part of the actual population *N* (${z}_{i}=1$) or not (${z}_{i}=0$) is derived from the inclusion probability Ψ with ${z}_{i} \sim {{{{{{\rm{Bernoulli}}}}}}}(\varPsi )$. Estimates of population size N (abundance) within each study area can then be obtained by summing all *z* values. Average density estimates can be derived by relating this number to the size of the respective area of interest. Further, sex was treated as a latent binary variable (female = 0, male = 1) allowing sex-assignment of unobserved animals and observed individuals with unknown sex based on a Bernoulli process. Detailed model description can be found in Supplementary Note 1.

### Statistics and Reproducibility

All analyses were performed using the *R* programming environment^88^ (Version 3.6.2). SCR models were fitted using the package *nimbleSCR*^89,90^ and the Bayesian modeling framework *nimble*^91^ (Version 0.12.0). We carried out reversible jump Markov-chain Monte Carlo sampling^92^ (rjMCMC) which enables proper sampling of the entire model and parameter space as well as convergence when using indicator variables^82,83^. The efficiency of parameter estimation was further increased by applying recent implementations of SCR functions in *nimbleSCR,* such as the local evaluation of the state space^90,93^. We ran four independent chains with 80 000 iterations including 20 000 iterations as burn-in. Every third sample was taken from the rjMCMC chains, resulting in a total of 80,000 posterior samples per parameter. To reduce memory usage, every tenth iteration only was retained (total of 30,000 samples) for estimates of AC locations. All parameters were checked for proper convergence either using the Gelman-Rubin diagnostics^94^ or visually for values impacted by the indicator variable^95^. For parameter estimates, we report posterior means and 95% Bayesian credible intervals (CI). Posterior values for the coefficient estimates of the point process only took into account iterations in which they were included (indicator γ = 1). Further information on model implementation and priors used is reported in the supplement (Supplementary Note 2; Supplementary Table 6). Relevant R code and input data to reproduce the SCR analyses have been deposited in a publicly accessible repository^96^.

We performed a post-hoc analysis to compare the estimated number of individuals located within and outside of forested areas within each study area. For this, we derived a binary grid (250 m) distinguishing forested from open landscapes from a landcover raster^81^. We grouped posterior samples of the AC placements from both SCR models based on the grid values and calculated sex-specific abundances and densities depending on the proportion of forested areas in each study area.

### Reporting summary

Further information on research design is available in the Nature Portfolio Reporting Summary linked to this article.

### Supplementary information

Supplementary Information

Description of Additional Supplementary Files

Supplementary Data 1

Supplementary Software 1

Reporting Summary

## Data Availability

The datasets necessary to reproduce analyses applied in this study are provided on Zenodo (10.5281/zenodo.8245739) and are publicly available. Raw data for reproducing Figs. 1 and 2 as well as Supplementary Fig. 2 are provided in the file “Supplementary Data 1”.

## Code availability

Code to reproduce analyses applied in this study is provided on Zenodo (10.5281/zenodo.8245739). R code for reproducing Figs. 1 and 2 as well as Supplementary Fig. 2 are provided in the file “Supplementary Software 1”.
