# Supplementary material for: Sexual segregation results in pronounced sex-specific density gradients in the mountain ungulate, Rupicapra rupicapra
Source: Commun Biol. 2023 Sep 25;6:979. doi: 10.1038/s42003-023-05313-z (PMC10520025; doi:10.1038/s42003-023-05313-z)
Supplement: Supplementary file 1 — Supplementary Information [file 42003_2023_5313_MOESM1_ESM.pdf]

Supplementary Information

Sexual segregation results in pronounced sex-specific density gradients in the mountain ungulate, *Rupicapra rupicapra*

Edelhoff, H.; Milleret, C.; Ebert, C.; Dupont, P.; Kudernatsch, T.; Zollner, A.; Bischof, R. & Peters, W.

Nature Communications Biology

Supplementary Table 1. Summary of individual chamois detected from successfully genotyped faecal samples. Overall and sex-specific results (Group) are reported for the two study areas including the number of detections, mean recapture rate, mean number of spatial recaptures (detections reprojected to the detector grid), and mean maximum distance moved (MMDM; values reported in metres) between spatial recaptures. Total numbers of detections deviate from reported numbers of genotypes as multiple detections of same individual within sub-detector grids are reduced to single detection.

Study Area	Group	Detected Individuals	Detections	Recapture Rate	Spatial Recaptures	MMDM
Chiemgau	Female	102	162	1.59	1.50	486
	Male	52	69	1.33	1.27	435
	Overall	154	231	1.50	1.42	472
Karwendel	Female	301	495	1.64	1.56	348
	Male	292	512	1.75	1.60	224
	Not Identified	23	24	1.04	1.04	527
	Overall	616	1031	1.67	1.56	286

Supplementary Table 2. Summary of posterior samples for the main parameters of the detection process from the two sex-specific SCR models for the two study areas. Includes posterior mean and 95 % credible intervals (CI). Values for σ are reported in meters.

Study Area	Parameter	Posterior mean	CI low	CI high
Karwendel	$p_{0,\ female}$	0.013	0.010	0.015
	$p_{0,male}$	0.024	0.021	0.029
	σ_{female}	227.1	211.1	244.3
	σ_{male}	149.5	139.5	160.5
	β_{search}	0.579	0.523	0.636
Chiemgau	$p_{0,\ female}$	0.005	0.004	0.007
	$p_{0,male}$	0.003	0.001	0.005
	σ_{female}	278.2	247.2	314.5
	σ_{male}	280.9	220.3	367.4
	β_{search}	0.631	0.528	0.736

Supplementary Table 3. Posterior Model Probabilities (PMP) of the point process component of the two SCR models. Variables (TRI, CANOPY, BARREN, SSI) included in each model (Null, 1, …, 15) are indicated by ones (included) and zeros (excluded). The model with highest posterior probability for each study area and sex is written in italics and highlighted in grey.

Sex	Model	TRI	CANOPY	BARREN	SSI	PMP (CG)	PMP (KW)
female	0	0	0	0	0	0.00	0.00
	1	1	0	0	0	0.00	0.00
	2	0	1	0	0	0.00	0.00
	3	1	1	0	0	0.34	0.33
	4	1	1	1	0	0.02	0.28
	5	0	0	1	0	0.00	0.00
	6	1	0	1	0	0.03	0.00
	7	0	1	1	0	0.00	0.00
	8	0	0	0	1	0.00	0.00
	9	1	0	0	1	0.00	0.00
	10	0	1	0	1	0.00	0.00
	11	0	0	1	1	0.00	0.00
	12	1	1	0	1	*0.44*	0.00
	13	1	0	1	1	0.02	0.00
	14	0	1	1	1	0.00	0.00
	15	1	1	1	1	0.15	*0.39*
male	0	0	0	0	0	0.00	0.00
	1	1	0	0	0	0.01	0.00
	2	0	1	0	0	0.00	0.00
	3	1	1	0	0	0.00	0.00
	4	1	1	1	0	0.00	0.00
	5	0	0	1	0	0.00	0.00
	6	1	0	1	0	0.00	0.00
	7	0	1	1	0	0.00	0.00
	8	0	0	0	1	0.00	0.00
	9	1	0	0	1	0.00	0.00
	10	0	1	0	1	0.00	0.00
	11	0	0	1	1	0.25	0.00
	12	1	1	0	1	*0.50*	0.00
	13	1	0	1	1	0.05	0.00
	14	0	1	1	1	0.03	0.00
	15	1	1	1	1	0.15	*1.00*

Supplementary Figure 1

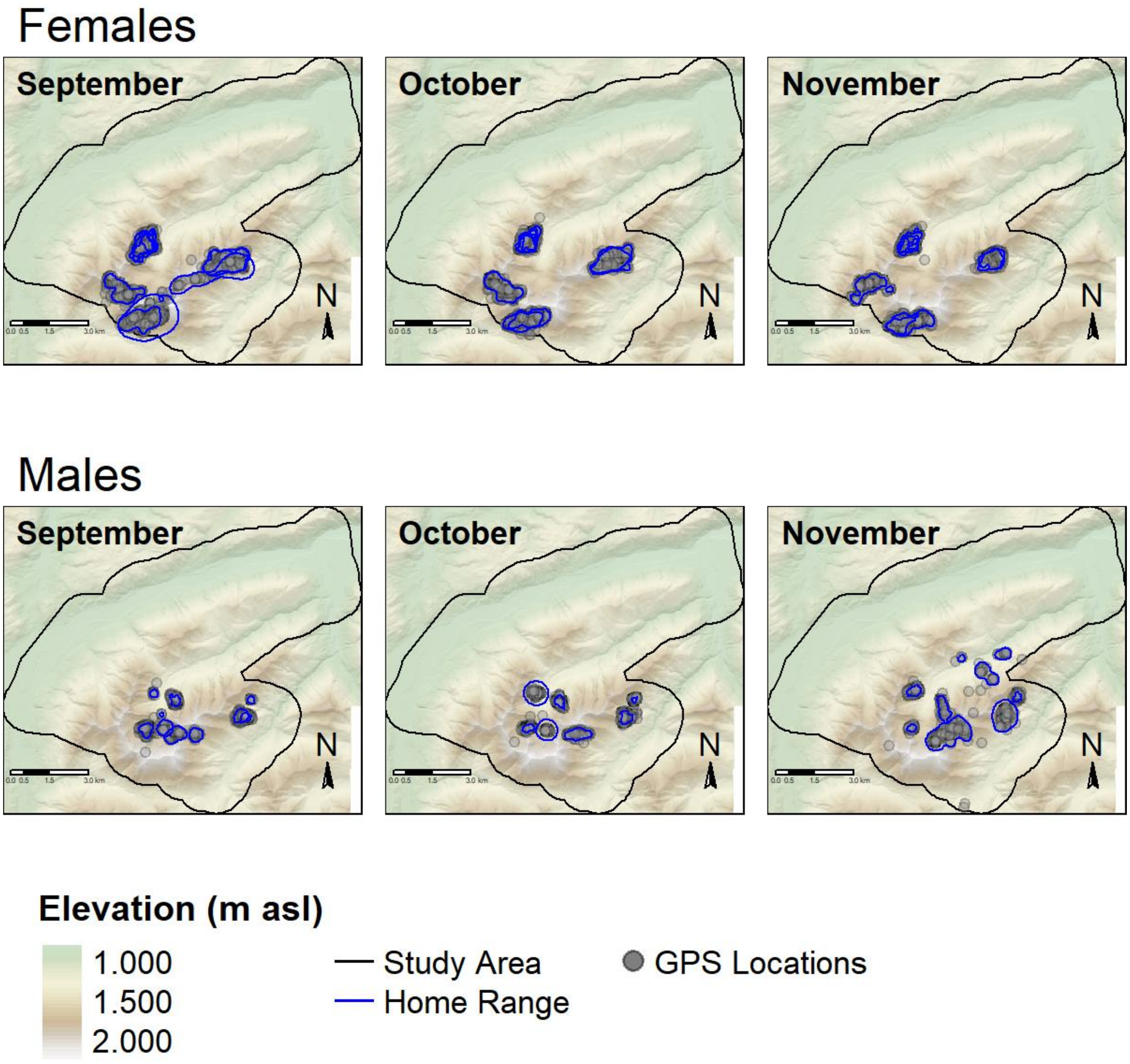

GPS telemetry locations of nine female (top) and seven male (bottom) chamois in the Karwendel study area. Observed relocations from 2019 and 2020 are shown for three months representing the respective autumn. Delineated home ranges are based on 95% kernel density estimates (blue lines). The study area was buffered by 1000m as in the SCR analysis of the main manuscript (black line).

Major changes in space use such as shifts of the home ranges are only observable for males in November, when faecal sampling was already completed.

Supplementary Note 1

The spatial capture-recapture (SCR) model employed in the main manuscript primarily estimates density and can therefore approximate habitat selection patterns at Johnson's[1] second-order (landscape) scale[2]. Here we aim to a) assess potential changes in habitat selection and space use by chamois during autumn, particularly before and during the rut; b) validate the effects of habitat covariates on density identified by SCR models with a second approach and independent data. We built resource selection functions (RSFs) at the second order scale using GPS chamois relocations. We employed a used-available design by comparing random points within chamois home ranges to available points within the buffered study area[1,3].

Telemetry Data

As part of another study, 16 chamois (9 females, 7 males) were fitted with GPS collars in the Karwendel study area. Chamois were captured using up-net traps, box traps, and aerial darting. Individuals were equipped with GPS radiocollars (Vectronics, Berlin, Germany) retrieving locations every two hours. Captures and data acquisition took place from spring 2019 to spring 2021 and were approved by the government of Upper Bavaria, Germany. We only considered telemetry data available for the two autumn periods ranging from first of September to November 30th for the resource selection analysis. Further, data were limited to match the buffered (1000m) study area of the SCR analysis resulting in data from 16 individuals (10 339 relocations in sum).

For each individual, monthly home ranges were derived with a 95 % kernel density estimator with reference bandwidth[4] implemented in the *amt* R package[5]. We sampled 500 random points within each home range representing habitat use. Available locations were sampled by distributing 2000 random points (equalling four times the used points) within the buffered

study area for each individual and each month (Supplementary Figure 1). For all locations, we extracted the values of the same habitat variables considered in the SCR analysis: the topographic terrain ruggedness index (TRI), the percentage of canopy cover (CANOPY), amount of barren ground (BARREN), and the site severity index (SSI). All habitat variable values were extracted from raster grids at 25m resolution.

Statistical Analysis

A resource selection function (RSF) estimates the relationship between habitat covariates and the probability of selecting a particular habitat unit[3]. Here, we applied logistic regression to quantify the influence of habitat covariates on resource selection, accounting for the binary nature of the response variable:

$$logit(p) = \beta_1 * TRI + \beta_2 * CANOPY + \beta_3 * SSI + \beta_4 * BARREN \quad (1)$$

with p describing the probability of a habitat unit being used (taking the value 1) or avoided (value of 0). Beta values (β_1, β_2, β_3, β_4) are the coefficients associated with the respective covariates TRI, CANOPY, SSI, BARREN.

We expected resource selection to be sex- and time-specific and further accounted for seasonal changes in selectivity by estimating RSFs for each sex and month (September, October, and November) separately. Maximum likelihood estimates of coefficients were obtained by fitting generalized linear models in R[6] with a "logit" link function and weighted data entries (used locations = 1; available locations = 0.25).

Results

With exception to SSI estimated coefficients were consistent across all fitted RSFs (Supplementary Figure 2). Coefficients for TRI were always positive and stronger than other model coefficients within all three months and for both sexes. In contrast, coefficients for

CANOPY were significant and strongly negative. The effect of BARREN was the weakest and negative in all RSFs. Site severity showed positive coefficients in females for all three months whereas the effect of SSI was slightly negative in September and November and non-significant in October in male chamois.

Habitat selection patterns obtained with RSFs and data from GPS collared individuals were comparable with the ones obtained with SCR models and non-invasive genetic sampling data (Supplementary Figure 2, Table 2 main text). The main differences concerned the variable SSI and CANOPY. Differences are expected between the SCR models and the RSFs. SCR models estimated the influence of habitat covariates on density and the RSFs estimated the strength of habitat selection from a few instrumented individuals. From the literature, we know that there is not always a clear relationship between density and habitat selection [7].

Supplementary Figure 2

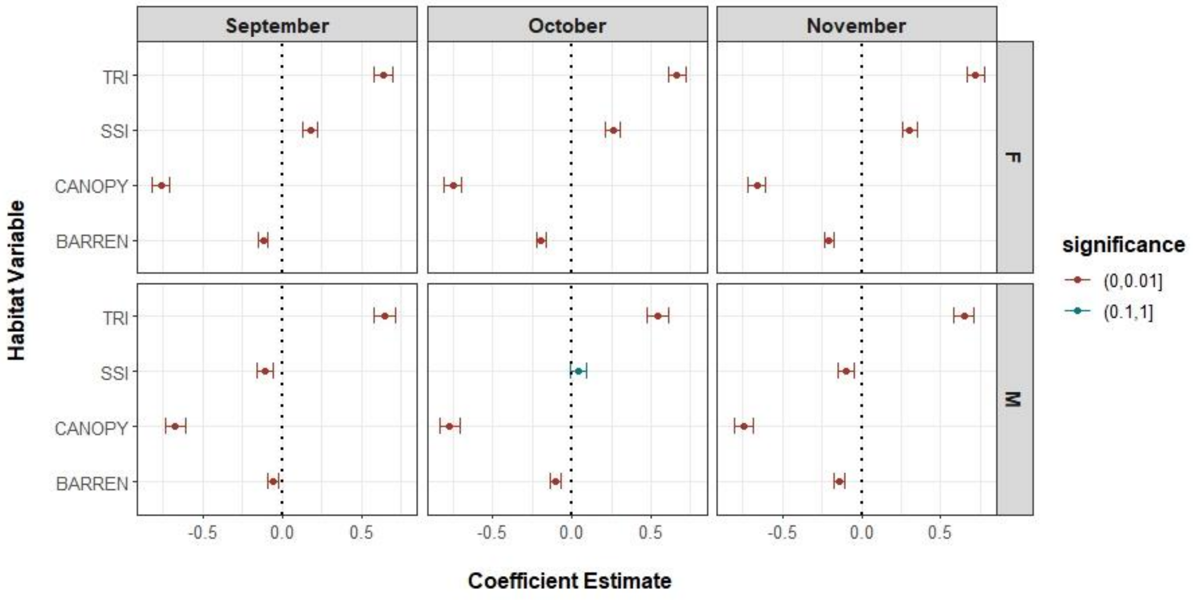

Coefficients of resource selection functions (RSF) fitted for female (top row, F) and male (bottom row, M) chamois in three different months of autumn. RSFs included all four habitat covariates also accounted for in the SCR model of the main study: terrain ruggedness (TRI), canopy cover (CANOPY), site severity (SSI), and amount of barren ground (BARREN). Estimates were derived using logistic regression comparing used and available habitat units. Error bars represent the 95% confidence intervals of each coefficient estimate.

Supplementary Table 4. Microsatellites used for individual identification and sex determination (marker AMELXY) of alpine chamois.

Locus	Size range (bp)	Number of alleles	Multiplex	Reference
HEL1	112 – 132	8	A	[19]
INRA36	176 – 188	7	B	[20]
OARFCB304	128 – 154	10	B	[21]
BM203	214 – 240	12	B	[19]
BM848	218 – 234	5	B	[19]
BMC1009	280 – 320	11	A	[19]
ETH225	134 – 160	8	A	[20]
SR-CRSP24	152 – 176	11	B	[22]
AMELXY	236 + 282	X+Y	A	[23]

Supplementary Table 5. Lower half of pairwise correlation matrix (Spearman's rank correlation test) of all covariates considered for the point process model in both study areas. Covariate values were derived from their respective input raster grids calculated separately for each study area. Correlation scores (*r* values) for the final covariates included in the model are highlighted in italic.

Study Area	Covariate	CANOPY	DEM	BARREN	TRI	SSI	BARREN_DIST	FOREST_DIST
Chiemgau	DEM	*.06*						
	BARREN	*.06*	.56					
	TRI	*.47*	.64	*.63*				
	SSI	*-.37*	.02	*.06*	*-.06*			
	BARREN_DIST	*-.14*	-.44	*-.88*	*-.56*	*-.11*		
	FOREST_DIST	*-.94*	.00	*-.05*	*-.46*	*.20*	.14	
	NDVI	*.67*	-.24	*-.26*	*.03*	*-.34*	.22	-.62
Karwendel	DEM	*-.31*						
	BARREN	*-.53*	.28					
	TRI	*-.15*	.80	*.27*				
	SSI	*-.46*	-.04	*-.05*	*-.11*			
	BARREN_DIST	*.46*	-.07	*-.94*	*-.09*	*.06*		
	FOREST_DIST	*-.92*	.34	*.59*	*.20*	*.31*	-.51	
	NDVI	*.84*	-.13	*-.55*	*-.05*	*-.32*	.52	-.83

Supplementary Figure 3

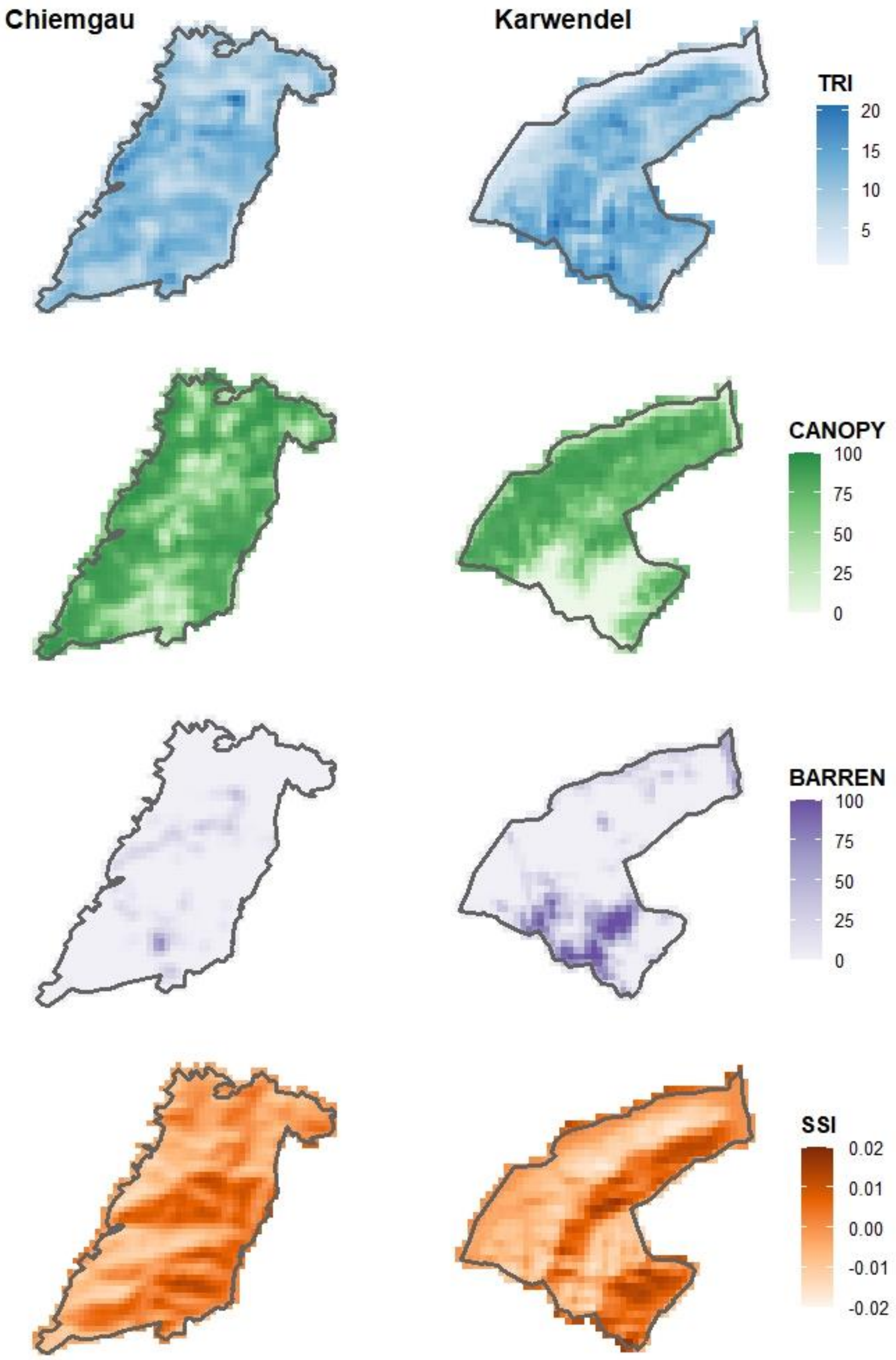

Spatial distribution of the four habitat covariates included in the ecological (point) process part of the SCR models. Maps show the topographic ruggedness index (TRI; no units,

increasing values indicate higher terrain ruggedness), percentage of canopy cover (CANOPY), percentage of barren landcover (BARREN), and site severity index (SSI; no units, low values indicate mesic sites and high values indicate xeric sites) within the Chiemgau (left column) and Karwendel (right column) study area.

Supplementary Note 2

Single-season Bayesian SCR models were fitted with sex-specific parameters to estimate chamois density for each study area[8,9].

Ecological / Point Process

We used an inhomogeneous Bernoulli point process[10] to model the influence of the habitat covariates on the distribution of ACs:

$$\log(I_{h,sex}) = \beta_{1,sex} \times TRI_h + \beta_{2,sex} \times CANOPY_h + \beta_{3,sex} \times BARREN_h + \beta_{4,sex} \times SSI_h \quad (2)$$

With I_h being the intensity of the point process in cell h and $\beta_{1,sex}$ to $\beta_{4,sex}$ the sex-specific coefficients associated with each of the four covariates: topographic ruggedness index (TRI[11]), canopy cover (CANOPY), amount of barren ground (BARREN), site severity index (SSI[12]).

To quantify the relative importance of covariates, we applied a Gibbs sampling procedure[13] and multiplied each coefficient estimate with a binary indicator variable (γ_1, γ_2, γ_3, γ_4):

$$\log(I_h) = \beta_{1,sex}\gamma_{1,sex} \times TRI_h + \beta_{2,sex}\gamma_{2,sex} \times CANOPY_h + \beta_{3,sex}\gamma_{3,sex} \times BARREN_h + \beta_{4,sex}\gamma_{4,sex} \times SSI_h \quad (3)$$

Depending on the value of the indicator, each covariate was either included (γ=1) or left out (γ=0) of the model for each posterior sample. This allows for simultaneous computation of posterior model probability (PMP), the proportion of times a given combination of covariates was included, and posterior inclusion probabilities (PIP[14]), the proportion of times a single variable was included in the model. Bayesian multimodel inference can then be performed accounting for all seven possible variable combinations. If none of the four covariates were

included, the model reduces to the null model and AC placement follows a uniform point process.

Detection Process

Conceptual traps were based on a detector grid of 100 m cell size over each study area[15,16]. Multiple detections of the same individual were partially aggregated into 50 m searched sub-grids allowing for up to four independent encounters (K) within one of the main detectors (PAB Model[17]).

Encounter frequency (y_{ij}) of an individual *i* at each detector *j* was assumed to follow a binomial distribution:

$$y_{ij} \sim BINOMIAL(p_{ij}, K_j) \tag{4}$$

with a maximum sample size of $K_j = 4$.

We modelled the decrease in detection probability (p_{ij}) with increasing distance d_{ij} between the AC of individual *i* and detector *j* based on the half-normal detection function[8]:

$$p_{ij} = p_{0\,sex_i j} \times \exp\left(\frac{-d_{ij}^2}{2\sigma_{sex_i}^2}\right) \tag{5}$$

Where p_{0j} is the detector-specific baseline detection probability at detector *j* and σ is a scaling parameter describing the level of decrease in the detection probability. When applying the half-normal detection function, σ is proportional to the radius of a circular individual activity area [8,18].

To account for non-uniform search effort among detectors, we used a generalized linear model formulation to estimate the detector- and sex-specific baseline detection probability ($p_{0_{sex_i} j}$):

$$logit\left(p_{0_{sex_i} j}\right) = \widehat{p_{0_{sex_i}}} + \beta_{search} B_j \tag{6}$$

With β_{search} being the coefficient describing the influence of the area searched within each detector grid cell B_j on the sex-specific baseline detection probability $\widehat{p_{0_{sex}}}$ (intercept). The

area searched effectively within each detector was approximated by placing a 3 m buffer around each search track to approximate the viewshed of two observers. Grid cells that were not searched were not considered.

We estimated the number of undetected individuals by data augmentation[8]. For each study area we set the maximum population size (*M*) to eight times the number of identified individuals throughout the corresponding field survey. Whether an individual i from M is also part of the actual population N ($z_i = 1$) or not ($z_i = 0$) is derived from the inclusion probability Ψ with $z_i \sim Bernoulli(\Psi)$. Estimates of population size N (abundance) within each study area can then be obtained by summing all *z* values. Average density estimates can be derived by relating this number to the size of the respective area of interest. Further, sex was treated as a latent binary variable (female = 0, male = 1) allowing sex-assignment of unobserved animals and observed individuals with unknown sex (Ψ_{sex}):

$$sex_i \sim Bernoulli(\Psi_{sex}) \qquad (7)$$

Supplementary Table 6. Information on priors used for parameters of the Bayesian SCR model including short description and applied *nimble* syntax.

**Parameter**	**Description**	**Prior**	**nimble implementation**
Ψ	inclusion probability of augmented individuals	uninformed, uniform distribution from 0 to 1	dunif(0, 1)
Ψ_{sex} (*sexratio* in R code)	probability of individual being male	uninformed, uniform distribution from 0 to 1	dunif(0, 1)
σ	scaling parameter of detection function	uninformed, uniform distribution from 0 to 50	dunif(0, 50)
p_0	baseline detection probability within detection process model	uninformed, uniform distribution from 0 to 1	dunif(0, 1)
β_{search} (*betaTRACKS* in R code)	coefficient of area searched in the detection process model	uninformed, uniform distribution from -5 to 5	dunif(-5, 5)
Ψ_{habcov} (*coeff_psi* in R code)	inclusion probability of habitat covariate in the point process model	uninformed, uniform distribution from 0 to 1	dunif(0, 1)
β_{habcov} (*habCoeff* in R code)	coefficients for habitat covariates of point process model	Uninformed, normal distribution (mean 0 and standard deviation of 10)	dnorm(0, sd=10)

Supplementary References

1. Johnson, D. H. The comparison of usage and availability measurements for evaluating resource preference. *Ecology* **61,** 65–71 (1980).
2. Royle, J. A., Fuller, A. K. & Sutherland, C. Unifying population and landscape ecology with spatial capture-recapture. *Ecography* **41,** 444–456; 10.1111/ecog.03170 (2018).
3. Manly, B. F. J., McDonald, L. L. & Thomas, D. L. *Resource Selection by Animals. Statistical design and analysis for field studies* (Springer Netherlands, Dordrecht, 1993).
4. Worton, B. J. Kernel Methods for Estimating the Utilization Distribution in Home-Range Studies. *Ecology* **70,** 164–168; 10.2307/1938423 (1989).
5. Signer, J., Fieberg, J. & Avgar, T. Animal movement tools (amt). R package for managing tracking data and conducting habitat selection analyses. *Ecol. Evol.* **9,** 880–890; 10.1002/ece3.4823 (2019).
6. R Core Team. *R: A language and environment for statistical computing* (2019).
7. Boyce, M. S. *et al.* REVIEW. Can habitat selection predict abundance? *J. Anim. Ecol.* **85,** 11–20; 10.1111/1365-2656.12359 (2016).
8. Royle, J. A., Chandler, R. B., Sollmann, R. & Gardner, B. *Spatial Capture-Recapture* (Elsevier Science, Burlington, 2013).
9. Borchers, D. A non-technical overview of spatially explicit capture–recapture models. *J. Ornithol.* **152,** 435–444; 10.1007/s10336-010-0583-z (2012).
10. Zhang, W. *et al.* A flexible and efficient Bayesian implementation of point process models for spatial capture-recapture data. *Ecology,* e3887; 10.1002/ecy.3887 (2022).
11. Riley, S. J., DeGloria, S. D. & Elliot, R. Index that quantifies topographic heterogeneity. *Intermountain Journal of Sciences* **5,** 23–27 (1999).
12. Nielsen, S. E. & Haney, A. Vegetation composition, dynamics, and management of a bracken-grassland and northern-dry forest ecosystem. *Environmental management* **31,** 810–821; 10.1007/s00267-003-0055-9 (2003).
13. O’Hara, R. B. & Sillanpää, M. J. A review of Bayesian variable selection methods. What, how and which. *Bayesian Anal.* **4,** 85–117; 10.1214/09-BA403 (2009).
14. Johnson, D. S. & Hoeting, J. A. Bayesian multimodel inference for geostatistical regression models. *PloS one* **6,** e25677; 10.1371/journal.pone.0025677 (2011).
15. Thompson, C. M., Royle, J. A. & Garner, J. D. A framework for inference about carnivore density from unstructured spatial sampling of scat using detector dogs. *J. Wildl. Manag.* **76,** 863–871; 10.1002/jwmg.317 (2012).
16. Russell, R. E. *et al.* Estimating abundance of mountain lions from unstructured spatial sampling. *J. Wildl. Manag.* **76,** 1551–1561; 10.1002/jwmg.412 (2012).
17. Milleret, C. *et al.* Using partial aggregation in spatial capture recapture. *Methods Ecol. Evol.* **9,** 1896–1907; 10.1111/2041-210X.13030 (2018).
18. Jennrich, R. I. & Turner, F. B. Measurement of non-circular home range. *J. Theor. Biol.* **22,** 227–237; 10.1016/0022-5193(69)90002-2 (1969).

19. Cassar, S., Galan, M. & Loison, A. A set of 21 polymorphic microsatellites in Alpine chamois (Rupicapra rupicapra). *Mol. Ecol. Notes* **7,** 243–247; 10.1111/j.1471-8286.2006.01569.x (2007).

20. Pérez, T., Albornoz, J. & Domınguez, A. A panel of bovine and caprine microsatellites suitable as markers in chamois. *Anim. Genet.* **31,** 344–345; 10.1046/j.1365-2052.2000.00674.x (2000).

21. Soglia, D. *et al.* Development of two multiplex PCRs for microsatellite analysis in Alpine chamois (Rupicapra r. rupicapra). *Ital. J. Anim. Sci.* **4,** 61–63; 10.4081/ijas.2005.2s.61 (2005).

22. Maudet, C. *et al.* A standard set of polymorphic microsatellites for threatened mountain ungulates (Caprini, Artiodactyla). *Mol. Ecol. Notes* **4,** 49–55; 10.1046/j.1471-8286.2003.00563.x (2004).

23. Gurgul, A., Radko, A. & Słota, E. Characteristics of X- and Y-chromosome specific regions of the amelogenin gene and a PCR-based method for sex identification in red deer (Cervus elaphus). *Mol. Biol. Rep.* **37,** 2915–2918; 10.1007/s11033-009-9852-4 (2010).
