# Supplementary material for: Sexual segregation results in pronounced sex-specific density gradients in the mountain ungulate, Rupicapra rupicapra
Source: Commun Biol. 2023 Sep 25;6:979. doi: 10.1038/s42003-023-05313-z (PMC10520025; doi:10.1038/s42003-023-05313-z)
Supplement: Supplementary file 2 — Description of Additional Supplementary Files [file 42003_2023_5313_MOESM2_ESM.pdf]

Description of Additional Supplementary Files

File name: Supplementary Data 1
Description: All required source data behind the graphs in the paper.

File name: Supplementary Software 1
Description: all R code to reproduce figures of the paper as well as the additional figures and analyses from the supplement.
