# Supplementary material for: Sexual segregation results in pronounced sex-specific density gradients in the mountain ungulate, Rupicapra rupicapra
Source: Commun Biol. 2023 Sep 25;6:979. doi: 10.1038/s42003-023-05313-z (PMC10520025; doi:10.1038/s42003-023-05313-z)
Supplement: Supplementary file 5 — Reporting Summary [file 42003_2023_5313_MOESM5_ESM.pdf]

nature portfolio

Corresponding author(s): Hendrik Edelhoff

Last updated by author(s): Aug 24, 2023

Reporting Summary

Nature Portfolio wishes to improve the reproducibility of the work that we publish. This form provides structure for consistency and transparency in reporting. For further information on Nature Portfolio policies, see our Editorial Policies and the Editorial Policy Checklist.

Statistics

For all statistical analyses, confirm that the following items are present in the figure legend, table legend, main text, or Methods section.

n/a	Confirmed	
☐	☒	The exact sample size (*n*) for each experimental group/condition, given as a discrete number and unit of measurement
☒	☐	A statement on whether measurements were taken from distinct samples or whether the same sample was measured repeatedly
☐	☒	The statistical test(s) used AND whether they are one- or two-sided<br>*Only common tests should be described solely by name; describe more complex techniques in the Methods section.*
☐	☒	A description of all covariates tested
☒	☐	A description of any assumptions or corrections, such as tests of normality and adjustment for multiple comparisons
☐	☒	A full description of the statistical parameters including central tendency (e.g. means) or other basic estimates (e.g. regression coefficient) AND variation (e.g. standard deviation) or associated estimates of uncertainty (e.g. confidence intervals)
☒	☐	For null hypothesis testing, the test statistic (e.g. *F*, *t*, *r*) with confidence intervals, effect sizes, degrees of freedom and *P* value noted<br>*Give P values as exact values whenever suitable.*
☐	☒	For Bayesian analysis, information on the choice of priors and Markov chain Monte Carlo settings
☐	☒	For hierarchical and complex designs, identification of the appropriate level for tests and full reporting of outcomes
☒	☐	Estimates of effect sizes (e.g. Cohen's *d*, Pearson's *r*), indicating how they were calculated

Our web collection on statistics for biologists contains articles on many of the points above.

Software and code

Policy information about availability of computer code

Data collection	All analyses are based on non-invasive genetic samples obtained from fresh chamois faeces collected in the field. For all faecal samples GPS coordinates were noted. For isolation of DNA, we used a commercial kit (NucleoSpin Soil Kit, Macherey-Nagel, Baesweiler, Germany). Amplification was performed using the Qiagen Multiplex PCR kit (Qiagen, Hilden, Germany). We determined allele sizes using the ABI GS500LIZ size ladder (Applied Biosystems, Darmstadt, Germany). Determination of matching genotypes was carried out with GENECAP (Version 1.4.). To confirm the power of the used loci, we calculated the probability of identity (PID) and PID for siblings using GIMLET (Version 1.3.3). Detailed description of the entire genotyping procedure provided in the manuscript.
Data analysis	Data processing and analyses were performed using the R programming environment (Version 3.6.2; R Core Team 2019). Bayesian models were fitted using the package nimbleSCR (Version 0.1.3) and the Bayesian modelling framework nimble (Version 0.11.1). All relevant R Code and custom functions are publicly available from a remote repository (Zenodo: 10.5281/zenodo.8245739).

For manuscripts utilizing custom algorithms or software that are central to the research but not yet described in published literature, software must be made available to editors and reviewers. We strongly encourage code deposition in a community repository (e.g. GitHub). See the Nature Portfolio guidelines for submitting code & software for further information.

Data

Policy information about availability of data

All manuscripts must include a data availability statement. This statement should provide the following information, where applicable:

- Accession codes, unique identifiers, or web links for publicly available datasets
- A description of any restrictions on data availability
- For clinical datasets or third party data, please ensure that the statement adheres to our policy

The datasets necessary to reproduce analyses applied in this study are publicly available through Zenodo (10.5281/zenodo.8245739).

Human research participants

Policy information about studies involving human research participants and Sex and Gender in Research.

Reporting on sex and gender	Study does not focus on humans.
Population characteristics	Study does not focus on humans.
Recruitment	Study does not focus on humans.
Ethics oversight	Study does not focus on humans.

Note that full information on the approval of the study protocol must also be provided in the manuscript.

Field-specific reporting

Please select the one below that is the best fit for your research. If you are not sure, read the appropriate sections before making your selection.

☐ Life sciences ☐ Behavioural & social sciences ☒ Ecological, evolutionary & environmental sciences

For a reference copy of the document with all sections, see nature.com/documents/nr-reporting-summary-flat.pdf

Ecological, evolutionary & environmental sciences study design

All studies must disclose on these points even when the disclosure is negative.

Study description	We estimated sex-specific densities and distributions for Alpine chamois (Rupicapra rupicapra r.) in two study areas using non-invasive genetic sampling in conjunction with a Bayesian spatial capture-recapture (SCR) analysis. We modelled densities as function of terrain ruggedness, forest canopy cover, site severity (solar radiation) and proportion of barren ground. Due to behavioural sexual dimorphism in this species, we expected a sex effect on distribution patterns as well as the detection process. Detailed description on the modelling procedure is provided in the manuscript and supplementary material.
Research sample	The research sample comprises 1451 spatial faecal detections which were assigned to 770 Rupicapra rupicapra r. individuals (both sexes). Detections were obtained non-invasively from genotyped faeces collected in two study areas.
Sampling strategy	We superimposed a 200 m search grid onto each study area and performed searches for fresh faecal piles within each of these grid cells. This approach ensured a sufficiently high resolution of the detectors to obtain unique captures of chamois individuals and spatial recaptures. Searches were unstructured but effort in space and time was recorded continuously using hand-held GPS units. This allowed us to account for variable sampling intensity in the analysis (see next section).<br>Study design was informed by a preliminary simulation study comparing different sampling strategies. The simulation study indicated a minimum sample size of 300 detections given a sampling resolution of 200m by 200m.<br>Average detection rates of more than 1.5 detections (i. e. recaptures) per individual suggest sufficient sample sizes .
Data collection	We performed unstructured searches for fresh faecal piles within daily units composed of up to 16 of the 200m grid cells. Search teams comprised two trained field workers. Only fresh faeces were collected, and their spatial location recorded with GPS devices (mainly Garmin eTREX 10 and Garmin 65 series). All search teams were instructed to cover the assigned grid cells homogenously with comparable intensity, but dangerous and inaccessible terrain was excluded. All search tracks were recorded with GPS devices to later account for spatially heterogenous search effort in the analysis. Sampling protocols were recorded for each faecal sample including information on date, time, location, and degree of freshness. Faecal samples were stored in 50 ml falcon tubes frozen at the end of each sampling day at -20° C.
Timing and spatial scale	Sampling took place over a three-week period for each study area in autumn 2018 (September 24th until October 7th in Karwendel and October 4th to 29th in Chiemgau). With the exception of dangerous and inaccessible terrain, both study areas were searched for faecal samples in their entirety.
Data exclusions	DNA of faecal samples which failed to amplify or to produce unambiguous results for more than two loci were discarded. For samples

Data exclusions	with one or two missing loci, we re-checked raw data for plausibility in case of matching genotypes and excluded any sample matching with more than one genotype. We also excluded samples showing signs of cross-contamination (i. e., genotypes with more than two alleles) from further analyses.<br>Additionally, we evaluated the distribution of observed distances between individual detections for outliers of long distances to avoid violations of the assumption of closed populations due to potential dispersal or migratory movements. Recaptures with distances within the 99 % percentile (longer than 1 500 m) were removed.<br>None of the aforementioned data exclusions are expected to introduce systematic bias during analysis.
Reproducibility	Detailed description of the data processing steps and applied modelling procedure provided in the methods section of the manuscript and the supplementary material. In addition, input data and R code to reproduce the performed analyses will be provided via Zenodo (10.5281/zenodo.8245739) upon acceptance.
Randomization	Randomization was not applicable due to a non-experimental approach in this study (i.e., spatial modelling). Instead, we focused on comprehensive coverage and accurate tracking of search effort throughout the study area.
Blinding	Genetic analysis (genotype designation etc.) was blind to spatial and temporal information associated with individual samples. Blinding was otherwise not applicable due to non-experimental approach in this study.

Did the study involve field work? ☒ Yes ☐ No

Field work, collection and transport

Field conditions	Field surveys were performed during fall of 2018. Temperatures during that period averaged 10°C (minimum = 3°C; maximum = 18° C). Fieldwork was temporally paused during rain.
Location	The “Karwendel” study area covers approximately 5 250 ha and is located in the correspondent mountain range (Lat = 47.509182; Long = 11.360413). Elevation ranges 800 m to approximately 2 350 m above sea level. The “Chiemgau” study area, covers about 7 250 ha and elevation ranges from 600 m to roughly 1 800 m above sea level (Lat = 47.744731; Long = 12.360889).
Access & import/export	The study involved only non-invasive methods; no animals were captured or handled during the work. No permits were required to carry out the field work and obtain faeces samples but field work was coordinated with management authorities.
Disturbance	Potential impacts of the study on vulnerable species were discussed with experts prior to sampling and disturbance within study areas was considered as negligible.

Reporting for specific materials, systems and methods

We require information from authors about some types of materials, experimental systems and methods used in many studies. Here, indicate whether each material, system or method listed is relevant to your study. If you are not sure if a list item applies to your research, read the appropriate section before selecting a response.

Materials & experimental systems

n/a	Involved in the study	
☒	☐	Antibodies
☒	☐	Eukaryotic cell lines
☒	☐	Palaeontology and archaeology
☐	☒	Animals and other organisms
☒	☐	Clinical data
☒	☐	Dual use research of concern

Methods

n/a	Involved in the study	
☒	☐	ChIP-seq
☒	☐	Flow cytometry
☒	☐	MRI-based neuroimaging

Animals and other research organisms

Policy information about studies involving animals; ARRIVE guidelines recommended for reporting animal research, and Sex and Gender in Research

Laboratory animals	This study did not include laboratory animals.
Wild animals	This study did not include wild animals, but non-invasive sampling of faecal pellets of wild animals.
Reporting on sex	Sex was considered as a covariate throughout the applied analyses and we derived both sex-specific detection probabilities as well as densities for each study area. Influence (strength and direction) of habitat covariates on density distribution was also estimated separately for each sex and study area.<br>Sex was derived from genetic samples using a x- and y-chromosome-specific region of the Amelogenin gene according to Gurgul et al.

	2010. In total, detections were assigned to 403 female and 344 male chamois respectively. In the case of 23 individuals sex could not be determined from the DNA samples.
Field-collected samples	Sampling protocols were recorded for each faeces sample including information on date, time, location, and degree of freshness. Faecal samples were stored in 50 ml falcon tubes frozen at the end of each sampling day at -20° C.
Ethics oversight	No ethical approval was required as the study only involved non-invasive genetic samples from faeces and no direct capture or handling of wild animals.

Note that full information on the approval of the study protocol must also be provided in the manuscript.
